# OSH Performance within TQM Application in Construction Companies: A Qualitative Study in Saudi Arabia

**DOI:** 10.3390/ijerph191912299

**Published:** 2022-09-28

**Authors:** Mohammed Alghaseb, Tariq Alshmlani

**Affiliations:** 1 Department of Architectural Engineering, College of Engineering, University of Ha’il, Ha’il 81451, Saudi Arabia; 2 Quality Engineering and Management, College of Engineering, University of Ha’il, Ha’il 81451, Saudi Arabia

**Keywords:** OSH, TQM, TBL, construction, Saudi Arabia

## Abstract

OSH plays a significant role in construction project success. Therefore, the aim of this study is to explore the influence of total quality management (TQM) application in improving occupational safety and health (OSH) within the context of Saudi construction companies. Factors were identified from structured literature reviews of previous relevant empirical studies. Then, these factors were theoretically framed into the concept of a triple bottom line (TBL), which includes three main dimensions: social, environmental, and economic. Thus, a semistructured interview survey was used to investigate these factors to address the performance of OSH in construction companies that implement TQM. A grounded theory was used to analyze and determine these factors. Accordingly, fourteen effective factors are identified. The survey findings indicate that the most influenced factors are the control of occupational accidents/injuries, the enhancement of workforce safety, the improvement of management pledges toward OSH, and the development of work culture toward OSH. These findings are vital in exploring the influence of TQM application in Saudi construction companies for the management of improving the performance of OSH, thereby helping to reduce the level of work injuries in the construction field and boosting the safety and health of workers for construction projects.

## 1. Introduction

The Saudi construction industry is considered a significant indicator of trends in the health of the national economy [1], being the largest recipient of government expenditure during the first three national development plans from 1970 to 1985 and exceeding 49% of the total government expenditures [2]. The significance of the construction industry includes the delivery of the infrastructure needed by other industries, reflecting the country’s economic development level [3]. Hence, the construction industry is considered to be amongst the most dangerous industries worldwide, in which the relationship between construction projects and safety risks and hazards shows a positive correlation [4,5]. Thus, the rate of accidents and deaths continues to increase due to continuous rises in the production of the construction industry [4], with high levels of accidents and death ratios in relation to other industries [6]. Thus, onsite safety involves ensuring the security of workers’ lives, making its implementation essential in construction projects [7]. Proceedings display that the number of occupational injuries in the Saudi construction industry between the years 2015 and 2020 was 17,299 cases with statuses including under treatment, cured, disability, and death [8]. Catastrophic accidents happen due to the underestimation of safety risks and the poor recognition of hazards [9]. Safety risks can be prevented through the presence of safety practices, procedures, and guidelines as essential elements for the survival and wellbeing of construction industry personnel. Still, the development of occupational safety and health principles from an organizational standpoint is a major focus of worldwide concern [10]. Therefore, both regulators and construction firms should arrange and verify the awareness of safety through implementation on sites [6]. Kilmacka presented the interpretation of the principles of occupational safety and health in the approach of quality management principles, which was the theoretical basis of the research [11].

Total quality management (TQM) is broadly perceived as a performance-improving program [12]. Deming defined quality as conformance to specification and being free from deficiencies [13]. Increasingly, economic and market globalization has indicated the important role of TQM and its associated standards [14]. Further, quality is an inclusive concept that embraces entire organizational features such as the involvement of all staff members in continuous improvement and the integration the quality principles into organizational culture [15]. TQM has been considered to be one of the major tools in management practice over the last decade [16] in manufacturing and service industries [17], as well as its benefits to the construction industry [18]. Implementing TQM can promote quality as a whole and improve business performance [19]. All improvement actions applied at firms to improve development are either direct (e.g., process organization) or indirect (e.g., work safety) [20]. Much research has been performed regarding the implementation of TQM; it is perceived that the benefits include higher customer satisfaction, better quality products, and higher market shares by construction companies [17]. Iqbal and Asrar-ul-Haq studied the relationship between TQM practices and employee performance in a dynamic technological region in Pakistan [21]. Amin et al. showed that TQM concepts had significant relationships with employee satisfaction [22]. In addition, Panuwatwanich found a significant influence of TQM on the performance of Vietnamese construction firms [18]. Goetsch and Davis stated that “total quality in reference with is an approach to doing business that attempts to maximize the competitiveness of an organization through the continuous improvement of the quality of its products, services, people, processes, and environments”, for which TQM contained eleven critical key elements [23]. Most of all, TQM definitions include a reference that is applied to its “soft” and “hard” sides [24], in which the “soft” side is related to elements such as leadership, employee empowerment, training and education, employee involvement, and teamwork [25,26]. The soft elements of TQM concern factors that directly affect people and may come from other soft elements [26]. Kilmacka and Matevz assessed the level of occupational health and safety based on the basic principles of quality management interpreted by [27]; therefore, the key principles of TQM used in this research are customer orientation, leadership, employee involvement, process approach, system approach to quality management, continuous improvement, and determining decisions based on facts. Then, these key principles are theoretically framed by the TBL.

The triple bottom line (TBL) is a philosophy that has dimensions representing sustainable development and is recognized and identified by interlinked interactions at three major dimensions, including the environment (planet), society (people), and economy (profit) [28]. The implementation of the TBL has been a successful approach for the sustainable development of firms, promoting economic growth and supporting environmental and social awareness [29]. Amponsah-Tawiah interpreted sustainable development as utilizing resources ideally in all respects [30]. The simultaneous integration of these three elements is required to determine sustainable development in the built environment effectively [31]. Therefore, the construction industry can be considered one of the most notable in sustainable development because it has a crucial influence on the TBL [32], and is critical to the sustainable development framework, affecting the three dimensions of the TBL: social, economic, and environmental [33]. The social dimension involves community participation, fair wages, and employee relations, with emphasis on the interaction between the firm in question and society [34]. The social dimension indicates the necessity for implementing beneficial and fair practices for work, society, and human capital, providing value to society, such as health insurance and fair wages [35]. The environmental dimension directly follows the generation of practices that preserve environmental resources from hazards, where environmentally sustainable firms should be practicing sustainability, such as the safe disposal of toxic waste [36]. Lastly, the economic dimension represents the impact of firm work practices on the economic system [37], emphasizing the economic value that the firm provides to surrounding systems [34]. Akanmu et al. revealed the significance of implementing TQM practices in organizations, considering it a key factor for increasing sustainability and achieving competitive advantage in the food industry [38], as well as Ho, who validated sustainable development through TQM [39].

Despite the advantage gained from the application of TQM in construction companies, the influence of TQM on OSH in the Saudi construction industry appears uncertain. In addition, TQM benefits to the construction industry are still unclear [40]. The Saudi construction industry is one of the sectors in which work injuries happen in abundance, recording the highest percentage of work injuries in the Saudi labor market. This sector has accounted for nearly 50% of work-related injuries in the last five years, and awareness plays a key role in promoting interest in OSH systems and practices. The most common risk factors affecting OSH in the Saudi construction industry are falling from a height, collisions of moving equipment, things falling, the collapse of trenches and excavations, scaffolding collapses, rubs or scrapes, and heat [41]. The research covering the topic of OSH in a Saudi construction context includes the study by Haadir and Panuwatwanich, who identified the critical factors affecting the successful implementation of safety programs [42], and that by Khasawneh, who investigated improving occupational health and workplace safety [43]. Mosely and Makki revealed the factors influencing safety climate perceptions [44] and explored the key components of determinants for the evaluation of the safety climate [45]. Almasoud established the national OSH standards at his institute [46]. Furthermore, the Occupational Safety and Health Department at the HRSD developed guidelines for the construction industry to enhance the awareness of the importance of adhering to OSH practices to create a safe and healthy work environment to save lives, property, and the environment from risks surrounding the work environment and to reduce the rate of work-related injuries and deaths [41].

Sanni-Anibire et al. investigated construction workers’ perceptions regarding OSH conditions in the eastern province of Saudi Arabia. The highest performing categories were the safety support climate, safety support activities, and organization climate, while management involvement, employee, and supervisor participation were the lowest performing categories [47]. Additionally, Erogul and Alyami explored construction workers’ perceptions regarding the construction site safety climate in Najran, the southern province of Saudi Arabia. The results revealed a lack of adherence to occupational health and safety regulations by employers, a need for construction site safety protocols and enhanced external inspection systems, an unawareness among participants in regard to the safety measures endorsed by their companies, and indications of leniency due to favoritism by external inspectors [48]. Moosa et al. investigated the factors causing accidents in Saudi Arabian construction companies. The three most important factors of poor safety performance were the firms’ top leaders, lack of training, and the reckless operation of equipment [49]. Thus, the Ministry of Municipal and Rural Affairs and Housing (MOMRAH) issued the Classification Points Program (CPP) in order to develop and improve services in the construction sector. The objective of the CPP is to monitor the quality of the sector’s work, divide construction sector facilities, raise compliance with performance quality standards, and follow up on the extent to which the facility classification scores are in line with the project implementation by linking field violations to the points program system [50]. The SBP has an instruction manual to service the quality inspection of contractors’ commitment to classification. The instruction manual has checklists that include general safety requirements, safety requirements in the workplace, site security requirements, environmental safety requirements, and occupational health requirements [51].

Nevertheless, the literature review indicates that limited work has thus far covered OSH in construction companies implementing TQM in the context of Saudi Arabia. Endeavors to improve OSH in construction companies by applying managerial philosophy such as TQM would facilitate governmental initiatives, thereby becoming a role model for different sizes and types of construction companies. The purpose of this study was to explore the influence of total quality management (TQM) application on improving occupational safety and health (OSH) within the context of Saudi construction companies.

## 2. Materials and Methods

**Figure 1:**
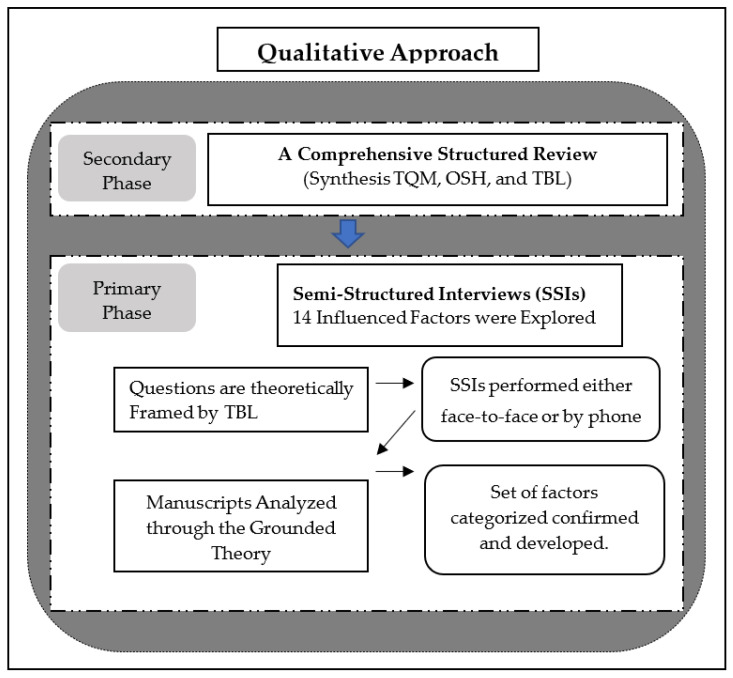
Research design (source: own study).

The authors used a qualitative approach of a combination of primary and secondary sources, as illustrated in Figure 1. The secondary source was a comprehensive structured review regarding OSH in construction, TQM, and the TBL. The primary source was semistructured interviews (SSIs) as shown in Appendix A. The structured review included synthesizing relevant research topics, including TQM, OSH, and sustainable development concepts in the form of the triple bottom line (TBL) framework.

**Figure 2:**
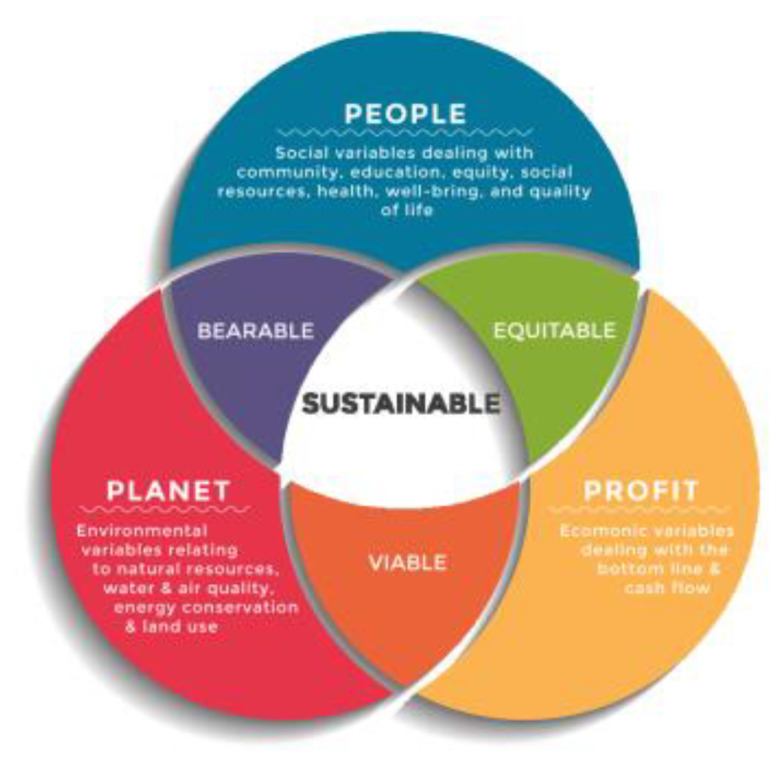
Triple bottom line [37].

**Figure 3:**
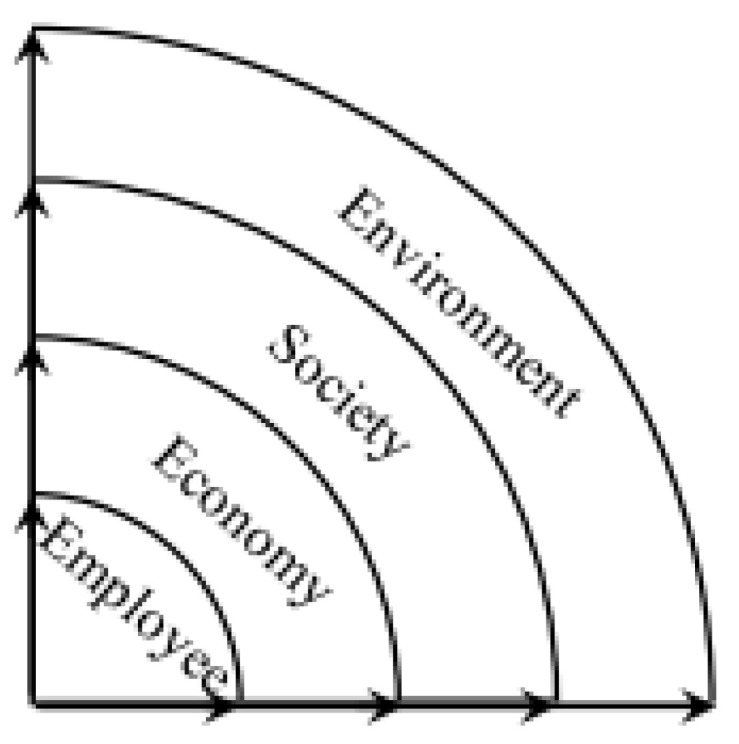
Approach of OSH and environment [28].

Figure 2 depicts the framework of the triple bottom line (TBL) approach. Figure 3 depicts a schematic framework of the viewpoints of the occupational, safety, health, and environmental aspects. Interviews are a common tool for gathering data and are very useful for quantitative question studies that have been widely used in the built environment [52]. Interviews include the use of open-ended questions or topics designed before data are collected; thus, SSIs are considered one of the most effective and convenient methods of collecting qualitative scientific data [53].

Further, data regarding work injuries were gathered from the Saudi Contracting Authorities [8]; Table 1 shows the distribution of injuries in the Saudi construction industry in the last five years, including the classification of injury types and revealing the decrease in work site injuries since 2016.

Table 2 shows the load of each injury number according to company type, including large, medium, and small.

However, the relevant research regarding quality management and safety management in manufacturing and service industries [11,27] revealed that seven principles of quality management can be converted into the context of occupational safety and health management, as shown in Table 3. Further, Panuwatwanich and Nguyen [18] investigated the Vietnamese construction context based on seven principles of TQM, including leadership management, training, employee relations, quality data and reporting, supplier quality management, project design, and process management [18]. Thus, the seven principles of TQM were used in the formation of the OSH management principles. Thus, this study was limited to the seven principles of TQM that were applied earlier in relevant construction safety studies, as shown in Table 3.

Occupational safety and health factors in construction industries have been reviewed, including the Saudi construction industry. Mosely reviewed and described eighteen factors from the previous literature that influenced the safety climate at construction project sites [6]. This study divided the OSH factors into TQM principles, then adopted the (TBL) approach as the theoretical framework. Srivastava et al. used the TBL to assess sustainability in construction projects. Section A in the SSI includes questions regarding company specialties, TQM applications, levels of TQM applications, types of OSH standards applied, and levels of annual work injuries. Section B presents semistructured interview questions on OSH factors with regard to social, environmental, and economical factors [54].

The authors of this study applied the exploratory method approach. The grounded theory is often heralded as revolutionary in the history of qualitative traditions [55]; therefore, it was used to analyze the manuscripts of interviews. A context analysis based on the constructivist grounded theory was performed using Nvivo software. The first level of coding was sorting the data to categorize the codes and generate themes based on the relationships between the codes, code frequencies, and meanings across the codes. Further, the second level of coding contained focused coding, axial coding, and theoretical coding. Examination of the initial codes was performed consistently with the identifying relationships. For the identification of the most frequent or significant initial codes, focused coding was applied. Axial coding was used to identify the core category and related categories. Ultimately, theoretical coding was used to connect the core category and related categories to create a storyline that should explain the phenomenon in terms of the thematic factors, as shown in Table 4.

A selection of indicator interviewees was considered. Technically, construction safety and health is mainly related to general stakeholder contractors. However, this research focused on the perception of general contractors in regard to measuring the influence of TQM applications on OSH in the context of Saudi Arabia. Thus, 32 firms were invited to participate in this study, and 15 firms were confirmed to participate according to the TQM applications in their firms. The participants in the study included seven general contractors, four subcontractors, and four specialized subcontractors from Riyadh, Jeddah, and the eastern region of Saudi Arabia. The regions are considered the largest sectors that drive the majority of construction projects in Saudi Arabia. The interview details are shown in Table 4.

## 3. Results

**Figure 4:**
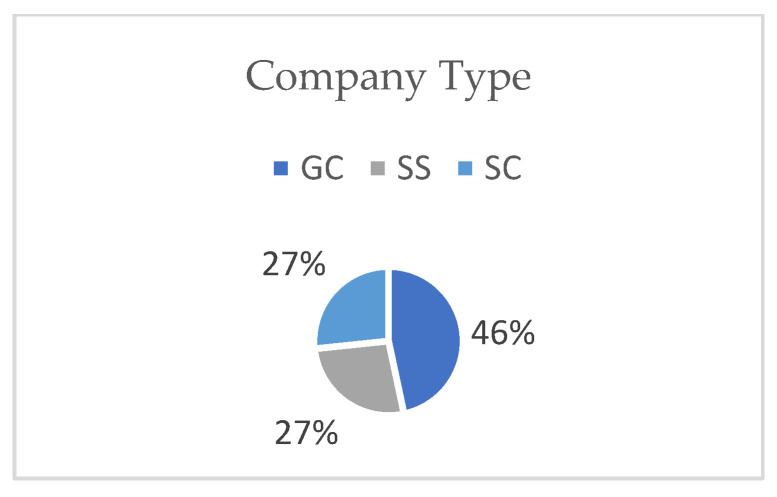
Company type (own study).

**Figure 5:**
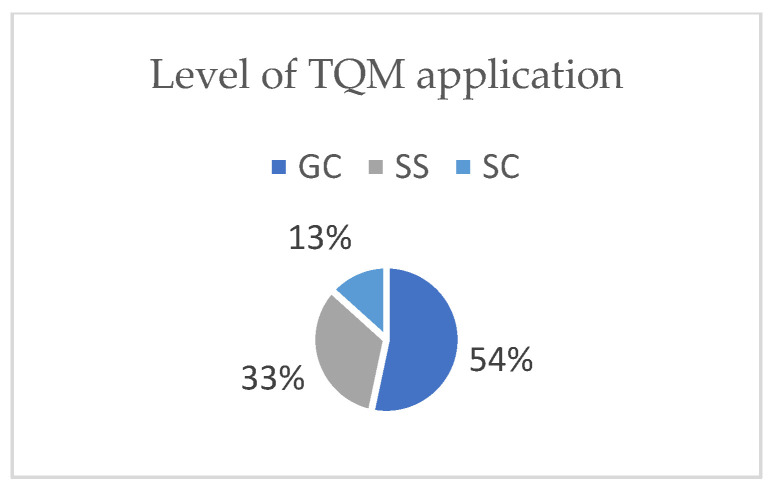
Level of TQM application (own study).

**Figure 6:**
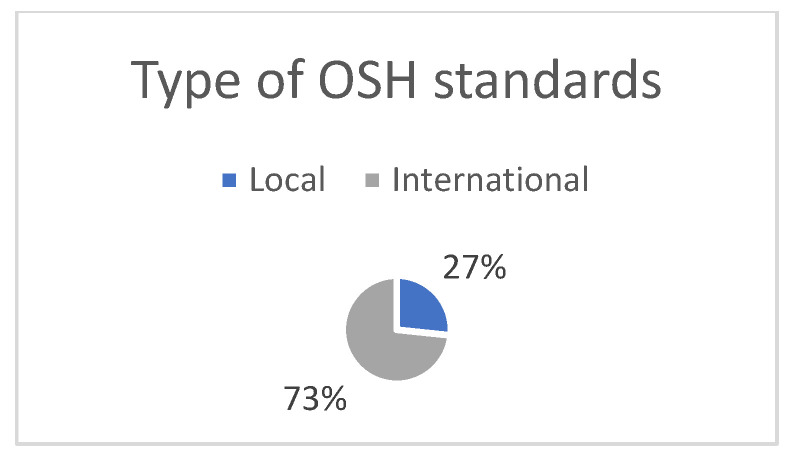
Type of OSH standards (own study).

**Figure 7:**
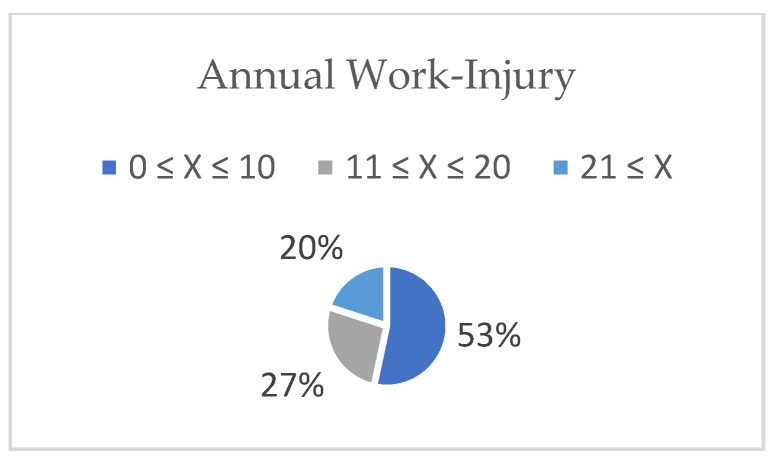
Annual injuries (own study).

As explained in the Section 2, the SSIs included two sections. Section A represented five aspects of TQM application. Participants who answered NO to the question regarding TQM application were excluded. Out of the thirty-two invitations to participate, and requiring experience in TQM, only fifteen participants in construction companies were willing to participate in this research. The participants included general contractors (46%), specialized subcontractors (27%), and subcontractors (27%), as shown in Figure 4. The levels of TQM application were similar, with 53% high, 34% medium, and 13% low, as shown in Figure 5. Further, the types of TQM standards used were local (27%) and international (73%) (Figure 6). Additionally, regarding the annual work injuries, 53% accounted for fewer than 10 cases, 27% between 11 and 20 cases, and 20% accounted for more than 21 cases, as shown in Figure 7.

The interviews with the practitioners focused on three themes—(I) social, (II) environmental, and (III) economical—as well as how these themes could be effective factors to ensure the influence of TQM application in OSH determinants within Saudi construction companies.

The participants represented three types of construction companies, including general contractors (GCs), specialized subcontractors (SSs), and subcontractors (SuBs). Table 5 shows the general backgrounds of the participants. The general contractor participants had the highest level of experience in total quality management with more than fifteen years, whereas specialized subcontractor and subcontractor participants had different ranges of experience with more than ten years.

**Figure 8:**
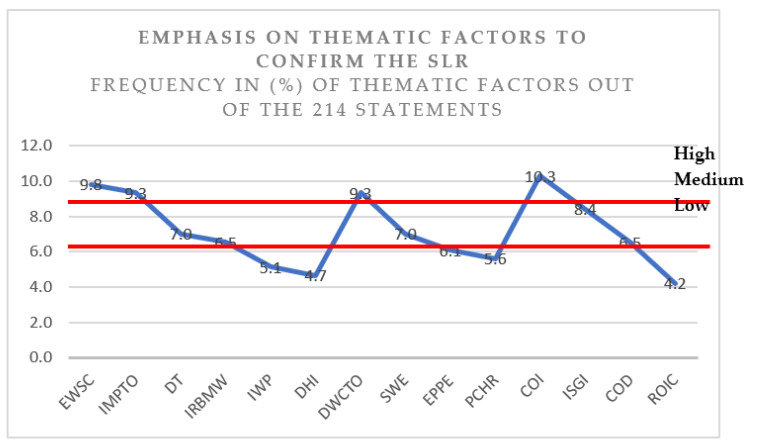
Cross-case analysis (source: own study).

Further, in a cross-case analysis, as illustrated in Figure 8, data from one interview were compared with those from another in order to identify the key similarities and differences and to allow patterns, trends, and relationships to emerge. Eisenhardt and Graebner explained the theories that emerged and were developed through the recognition of patterns of the relationships among the themes within and across interviews, as well as their fundamental logical arguments [56]. Therefore, the data were categorized as low, medium, and high based on their objective occurrence in the following domain scales, as shown in Table 6 and Figure 8.

The findings are presented in Table 7 and show that a major frequency was established. The thematic factors were categorized as well. The total frequency was 214, and each factor repetition was loaded depending on the total frequency to provide the percentage of each factor. Thus, six influenced social factors, four influenced environmental factors, and four influenced economic factors were reported, including the frequency, percentage of frequency, and scale depending on the cross-case analysis.

## 4. Discussion

As explained in the Section 2, the interviews with the practitioners focused on three themes—(I) social, (II) environmental, and (III) economical—as well as how these themes could be effective factors for ensuring the influence of TQM application in OSH determinants within Saudi construction companies. Out of the thirty-two invitations to participate, with required experience in TQM, only fifteen participants in construction companies were willing to participate in this research. The participants represented three types of construction companies, including general contractors (GCs), specialized subcontractors (SSs), and subcontractors (SuBs). Table 4 shows the general backgrounds of the participants. General contractor participants had the highest level of experience in total quality management, amounting to more than fifteen years, whereas specialized subcontractor and subcontractor participants had different ranges of experience, amounting to more than ten years.

### 4.1. Influenced Social Factors

Influenced social factors were fairly mentioned by participants. However, the transcripts revealed that the interviewees were well aware of the key role of TQM application in construction companies as influential drivers of the social factors of occupational safety and health. Therefore, the application of TQM tools and techniques, such as benchmarking and information technology, positively influenced the social factors. The influenced social factors included enhanced workforce safety, improved management pledges toward OSH, developed training, improved relationships between management and workforces, improved workforce participation, and developed health insurance.

#### 4.1.1. Enhanced Workforce Safety and Improved Management Pledges toward OSH

The highly significant influenced social factors pointed out by the interviewees were enhanced workforce safety (frequency: 9.8%) and improved management pledges toward OSH (9.3%). The participants mentioned enhanced workforce safety in different contexts, including construction and maintenance types of projects. Applying the total productive maintenance and management tools as TQM tools positively contributed to improving worker safety and commitment to OSH. As stated by interviewee MA: “One of the most important aspects of quality applications in projects is the improvement of the workers’ safety, whether inside the facility or outside in the construction site. It is also noticeable that the occupational safety tools are better applied inside the company’s buildings”. The perception of interviewees regarding “enhancing workforces’ safety” was consistent with [11], where quality management principles were converted into safety management, showing that employees of large enterprises in the service sector assessed the safety of work very highly and also demonstrated knowledge of the legal regulations in this area. Another consistent example is a study in China that identified six safety climate components, including the “safety environment” [57]. Otherwise, inconsistency appeared in [27] in manufacturing, where it was found that regardless of the size of the enterprise, employees had doubts about the priority of their safety. Nonetheless, large company respondents in the same study rated the factor “focus on employee safety” higher than in small and medium companies. In addition, the majority of participants pointed out the management’s commitment to OSH. As mentioned by interviewee HA: “The administration works to follow up on compliance with occupational safety and health standards on a regular basis, and reports are submitted in this regard to the officials”. This finding is consistent with [45], where “Management Commitment to Safety” was confirmed as a significant factor for safety climate evaluation in the Saudi construction industry. Additionally, the findings by [58] stated that the factors describing TQM implementation in service companies concerned “quality practices of top management”.

#### 4.1.2. Developed Training and Improved Relationships between Management and Workforces

In contrast, developed training (7.0%) and improved relationships between management and workforces (6.5%) were identified as moderately influenced social factors. These factors appeared to be moderate because of the managerial and financial inter-relating, where the financial department was also responsible for funding the training. As mentioned by interviewee MK: “Yes, we notice an improvement in the performance of quality in general through the commitment of managers towards it, but there are many questions that need answers regarding training workers and obtaining certificates in total quality management, especially for foreign workers”. This finding is supported by [59], in which safety training was identified as the most significant factor. Inconsistently, Durdyev et al. in Cambodia found that safety-related issues were not a priority of contractors [60], including regular training [61], while the human resources department was responsible for improving relationships among job structures, including obligations and terms, including duties in job descriptions. As mentioned by interviewee HA: “Indeed, total quality is used in the company, and employees are asked to respond to changes towards quality, and we notice a slight improvement in the relationship of employees with management”. This finding is consistent with [45], which found that interactions were significantly in the form of worker communication with other coworkers or management regarding safety aspects. Additionally, consistent with [62], it was found that people-related TQM practices were positively related to job satisfaction at both the individual and organizational levels. Inconsistently, Haadir and Panuwatwanich in Saudi Arabia found that insignificant and last-ranked factors were open communication between management and the middle-level and bottom-level staff reporting unsafe conditions [42].

#### 4.1.3. Improved Workforce Participation and Developed Health Insurance

The participants had dissimilar perceptions about the factors, somewhat stating that subcontractors and specialized subcontractors showed less awareness compared to general contractors. Further, improved workforce participation (5.1%) and the development of health insurance (4.7%) were somewhat stated as influenced social factors. The participants indicated that the lower worker involvement is because of a lack of awareness about TQM in the construction field. Additionally, most safety meetings were missed by subcontractors and specialized subcontractors. Workforce participation in TQM application reflected the employee involvement in the implementation of the mission, vision, and goals of safe working conditions. However, the general contractor perceptions, who were highly aware of TQM’s positive influence on workforce participation, were consistent with the concluded results in [18], which found that all seven measured variables, including “employee involvement”, were significantly related to the TQM construct in the Vietnamese construction industry. Inconsistently, the specialized subcontractor and subcontractor perceptions are supported by [61] and indicate that it is essential for the main stakeholders to concentrate on the most neglected aspects of OSH, including safety meetings and worker involvement. Additionally, the development of health insurance reflected the system approach to safety management. The participants somewhat mentioned improvements concerning insurance packages after applying TQM in their businesses. While health insurance is mandatory when contractors bid for contracts, developing health insurance packages was attributed to compulsory insurance for companies cooperatively introduced by “MOMRAH, the “Council of Health Insurance”, and the “Saudi Contactors Authority”. Moreover, this general contractor perception is supported by [63], in which the results of the literature review analysis stated that the ISO 45001 Occupational Health and Safety Management System had direct benefits, including reductions in insurance premium costs. Inconsistently, the subcontractor perceptions agreed with [64], which stated no insurance policy for the health of workers.

### 4.2. Influenced Environmental Factors

The influenced environmental factors were fairly mentioned by participants. However, the interviewees were well aware of the key role of TQM application through tools and techniques, such as environmental management systems, quality function development, and service liability in construction companies as influential drivers of environmental factors in occupational safety and health. However, the influenced environmental factors included the development of a work culture toward OSH, securing the working environment, enhanced personal protective equipment, and planned/controlled hazards and risks.

#### 4.2.1. Developed Work Culture toward OSH

In addition, the highly significantly influenced environmental factor that interviewees mentioned was a developed work culture toward OSH (9.3%). The majority of participants obviously indicated that employers seemed serious about applying the principles of TQM in their businesses. As mentioned by interviewee WA: “For decades, construction projects have been suffering from poor quality, which has caused many construction projects to be delayed. With the attention of the supervisory authorities to this shortcoming, the importance of developing a quality management department within the firm has grown among the major contracting companies. I believe that quality starts inside the organization”. This finding is consistent with [45], which indicated that worker attitudes toward health and safety represented a form of safety interaction. Additionally, according to [65], a workmate’s influence was a similar safety climate factor structure in OSH. Additionally, inconsistent with the results found in [60], it was revealed that the “lack of monitoring the compliance of safety measures” was a hindering factor in construction safety performance.

#### 4.2.2. Secured Working Environment

In contrast, a secured working environment (7.0%) was identified as a medium-influenced environmental factor. Therefore, it was obvious that factors that require financial commitment are still in progress; thus, the participants’ points of view are still in the implementation phase of the TQM journey inside the organization. This finding is supported by [47], which found thirteen factors influencing the safety climate, including a “supportive environment” in the construction industry of Saudi Arabia.

#### 4.2.3. Enhanced Personal Protective Equipment and Planned/Controlled Hazards and Risks

Further, enhanced personal protective equipment (6.1%) and planned and controlled hazards and risks (5.6%) were sometimes stated. However, changes require much knowledge regarding attention and financial capabilities to significantly achieve such factors. Ngwama supported this finding, where one of the key elements for effective trade union influence in OSH issues was “safety education” on worker requirements to wear personal protective devices to reduce their exposure to hazards [66]. In contrast, and inconsistent with this finding, [60] found that regarding the resources issue, the most influential safety items included a lack of personal protective equipment; thus, providing personal protective equipment would help avoid work-related fatalities and injuries. Further, planned and controlled hazards and risks as a finding are consistent with [44], which confirmed that the appraisal of risks and hazards as climate safety factors in the form of the ability to assess risks and hazards was present in the Saudi construction industry. On the other hand, Zahoor found that worker involvement in planning also needed special attention in developing countries, where workers were observed to be unconcerned about their safety and tended to take shortcuts and expose themselves to unnecessary risks [61]. The transcripts of the interviews revealed a lack of funding to execute planned work regarding TQM for protection from either onsite risks or hazardous materials. Onsite risks include all possible causes of work injuries, while hazardous materials need careful treatment, for instance, of chemical materials. The participant views were deemed to be minor regarding the outcome of TQM onsite. Thus, construction companies need time for executing obligations toward applying TQM on construction sites.

### 4.3. Influenced Economic Factors

Influenced economic factors were clearly mentioned, and the participants were well aware of the key role of TQM tools and techniques in construction companies. These tools include statistical process control, management tools, failure mode, and effect analysis as influential drivers of the economic factors in occupational safety and health. The influenced economic factors included controlled occupational injuries, controlled occupational diseases, improved supervision and inspection, and reduced occupational injury costs.

#### 4.3.1. Controlled Occupational Injuries

Although one of the highly significantly influenced economic factors pointed out by the interviewees was controlled occupational injuries (10.3%), the transcripts revealed that the interviewees were well aware of the OSH instructions required onsite, ascribing this to the extraordinary attention paid by MOMRAH through quality initiatives (e.g., the ranking points program and sustainable building program), which help prevent onsite injuries. Additionally, the majority of respondents reported much fewer work injuries than what was in the governmental authority’s annual reports, reflecting the efforts undertaken by the regulators. As mentioned by interviewee SQ: “A number of quality-related initiatives have been developed to reduce workplace injuries, which have actually contributed to reducing work-related injuries among workers in the construction sector”. In addition, statistical reports on the Saudi Contractors Authority website revealed decreasing injuries in construction sites since 2015. This finding, supported by [63,67], found that the main benefit pointed out by company respondents was the reduction in work-related accidents in Portugal. Additionally, Durdyev et al. [60] agreed with this finding in the discussion of the results, where detailed pre-construction planning [68] played a significant role in avoiding a tight schedule and overlapping activities and gaining benefits from these implementations, including fewer construction site accidents, as shown in Table 2.

#### 4.3.2. Controlled Occupational Diseases and Improved Supervision and Inspection

In contrast, controlled occupational diseases (8.4%) and improved supervision and inspection (6.5%) were identified as medium-influenced economic factors. Constructors were deemed to care less about other safety considerations, such as worker housing, where the human factor to spatial awareness was missing. Thus, regulators, such as the HRSD and the Public Health Authority, established the *Health Requirements for Worker Housing* during the COVID-19 pandemic in order to prevent and control diseases among workers, as well as the guide manuals for *Healthy and Safe Office Work Environments* and *Health and Safety in the Work Environment*. Moreover, few participants indicated that contractors refrained from projects that required many safety inspections. This finding is consistent with [67], since the benefits identified by companies that applied safety management systems included reducing occupational diseases and conducting regular audits and/or safety inspections on a regular basis. Our results inconsistently agree with [69], which found a lack of inspection procedures onsite, as well as [70], which also concluded that the overall impact of inspections on psychosocial risk management was still rather limited.

#### 4.3.3. Reduced Occupational Injury Costs

Eventually, reduced occupational injury costs (4.2%) were somewhat stated. The majority of participants seemed less aware of the cost–benefit gained from having few work injuries, since few participants mentioned it, as well as confirming controlling occupational injuries to be an economic factor. This finding is consistent with [67] in reducing the costs of accidents. On the other hand, it is inconsistent with [45], which claimed that many construction companies in Saudi Arabia tried to control the rising costs of accidents and reduce project delays due to accidents through safety programs.

A summary of the findings and interpretations of the TQM principles is shown in the last column in Table 8.

## 5. Conclusions

This study aimed to explore the influence of TQM on the performance of OSH in Saudi construction companies. An exploratory approach was used and OSH factors were determined based on semistructured interviews. This study found that the TBL principle was a valuable framework and novel approach to propose a beneficial exploration approach. Three main dimensions were quarried using the TBL philosophy, which included social, environmental, and economical aspects. There were six social, four environmental, and four economical influenced factors; the study concluded that these fourteen OSH factors were significantly influenced by the application of TQM in the Saudi construction industry. The highest influenced factors were the enhancement of workforce safety, the improvement of management pledges toward OSH, the development of work culture toward OSH, and the control of occupational injuries, while developing health insurance plans, controlling hazards and risks, and reducing occupational injury costs were the lowest performing factors. The OSH factors revealed in this study could help construction industry stakeholders to understand the advantages of TQM application in construction firms, thereby enhancing OSH performance on construction sites and reducing work-related injuries. The OSH factors found in this study resulted from a qualitative approach within the context used. Therefore, for future research, it is suggested to formulate the findings into propositions to generalize them into a quantitative questionnaire survey as well as the scope of the study, covering both consultant and contractor firms, which could provide more demonstrative data about the influence of TQM application on the performance of OSH in Saudi construction companies. Furthermore, dissimilarities in the interviewee perceptions appeared based on the sizes and types of the construction firms; thus, further research regarding the implementation stages of TQM in the Saudi construction industry would facilitate the construction of strategy roadmaps for stakeholders.

## Figures and Tables

**Table 1:** Injury numbers [8].

Distribution of Number by Injury Status	Years				
	2016	2017	2018	2019	2020
Under Treatment	4250	3969	2811	2688	3581
Cured	816	777	676	1134	69
Disability	489	513	461	180	27
Death	159	101	91	28	9
TOTAL	5714	5360	4039	4030	3686

**Table 2:** Company size [8].

Distribution of Number by Company Size	Years				
	2016	2017	2018	2019	2020
Large	4250	3969	2811	2688	2080
Medium	816	777	676	1134	914
Small	648	614	552	208	692
TOTAL	5714	5360	4039	4030	3686

**Table 3:** The principles of OSH in the approach to QM [11].

	Principles of Quality Management	Principles in the Context of Occupational Safety and Health Management
**PRINCIPLE 1**	Customer orientation	Focus on employee safety—putting employee needs above economic, efficiency, and productivity conditions
**PRINCIPLE 2**	Leadership	Being a role model in the aspect of care for work safety
**PRINCIPLE 3**	Employee involvement	Employee involvement in the implementation of the mission, vision and goals of safe working conditions
**PRINCIPLE 4**	Process approach	Documenting the inter-relationships of processes aimed at maintaining safety
**PRINCIPLE 5**	System approach to quality management	System approach to safety management
**PRINCIPLE 6**	Continuous improvement	Introducing additional safety systems, conducting regular training, etc.
**PRINCIPLE 7**	Determining decisions based on facts	Legal documents, statistics, and results of monitoring security parameters
**PRINCIPLE 8**	Mutual beneficial relationships with suppliers	Not applicable

**Table 4:** Interview schedule (N = 15) (source: own study).

Interviewee Identifier		Type	Interviewee Title	Location	Interview Type	Duration
**General Contractors**	**HD**	CE	QM Director	Riyadh	Phone	00:38:23
	**MS**	EE	QM Director	Riyadh	Office	00:59:31
	**NA**	ME	QM Senior Manager	Jeddah	Phone	00:47:32
	**AH**	CE	QM Site Engineer	Riyadh	On Site	01:03:39
	**MA**	CE	QM Site Engineer	Riyadh	On Site	01:13:16
	**MK**	ME	QM Manager	Riyadh	Office	00:48:57
	**WA**	EE	QM Senior Manager	Dammam	Phone	00:43:11
**Specialized Sub.**	**HA**	ME	QM Div. Manager	Riyadh	Office	00:59:16
	**SK**	ME	QM Div. Manager	Riyadh	Office	00:56:08
	**AM**	EE	QM Director	Riyadh	Phone	00:47:23
	**SQ**	CE	QM Director	Dammam	Phone	00:36:58
**Sub.**	**TA**	EE	QM Site Engineer	Riyadh	On Site	01:09:18
	**MD**	ME	QM Director	Jeddah	Phone	00:38:14
	**MC**	CE	QM Senior Manager	Riyadh	Office	00:51:39
	**KK**	EE	QM Director	Jeddah	Phone	00:42:18

**Table 5:** Participant expertise (source: own study).

	General Contractor (GC)							Specialized Subs. (SS)				Subcontractors ((SC)			
**Participant specialty**	CE	EE	ME	CE	CE	ME	EE	ME	ME	EE	CE	EE	ME	CE	EE
**TQM experience**	17	22	18	20	21	19	17	14	10	10	12	11	13	10	12

**Table 6:** Scale of importance (source: own study).

Scale of Importance		
**Low**	**Medium**	**High**
X < 6.5%	6.5% < X < 8.5%	X > 8.5%

**Table 7:** Research findings (source: own study).

Influenced OSH Factors	Thematic Factor	Freq.	Thematic Coding	Percentage	Scale
**Social**	Enhance workforce safety	21	EWSC	9.8%	High
	Improve management pledge toward OSH	20	IMPTO	9.3%	High
	Develop training	15	DT	7.0%	Medium
	Improve relationship between management and workforces	14	IRBMW	6.5%	Medium
	Improve workforce participation	11	IWP	5.1%	Low
	Develop health insurance	10	DHI	4.7%	Low
**Environmental**	Develop work culture toward OSH	20	DWCTO	9.3%	High
	Secure working environment	15	SWE	7.0%	Medium
	Enhance personal protective equipment	13	EPPE	6.1%	Low
	Plan and control hazards and risks	12	PCHR	5.6%	Low
**Economical**	Control occupational injuries	22	COI	10.3%	High
	Improve supervision and inspection	18	ISGI	8.4%	Medium
	Control occupational diseases	14	COD	6.5%	Medium
	Reduce occupational injuries costs	9	ROIC	4.2%	Low
**Total**		**214**			

**Table 8:** Findings linked to TQM principles (source: own study).

	Principles of Total Quality Management	Link Finding Factors to TQM Principles
**PRINCIPLE 1**	Customer orientation	Soc.:1; Env.:2
**PRINCIPLE 2**	Leadership	Soc.:2; Env.:1
**PRINCIPLE 3**	Employee involvement	Soc.:5
**PRINCIPLE 4**	Process approach	Soc.:4; Eco.:1; Eco.:2
**PRINCIPLE 5**	System approach to quality management	Soc.:6; Env.:3
**PRINCIPLE 6**	Continuous improvement	Soc.:3; Eco.4
**PRINCIPLE 7**	Determining decisions based on facts	Env.:4; Eco:3
**PRINCIPLE 8**	Mutual beneficial relationships with suppliers	Not applicable

**Figure A1:**
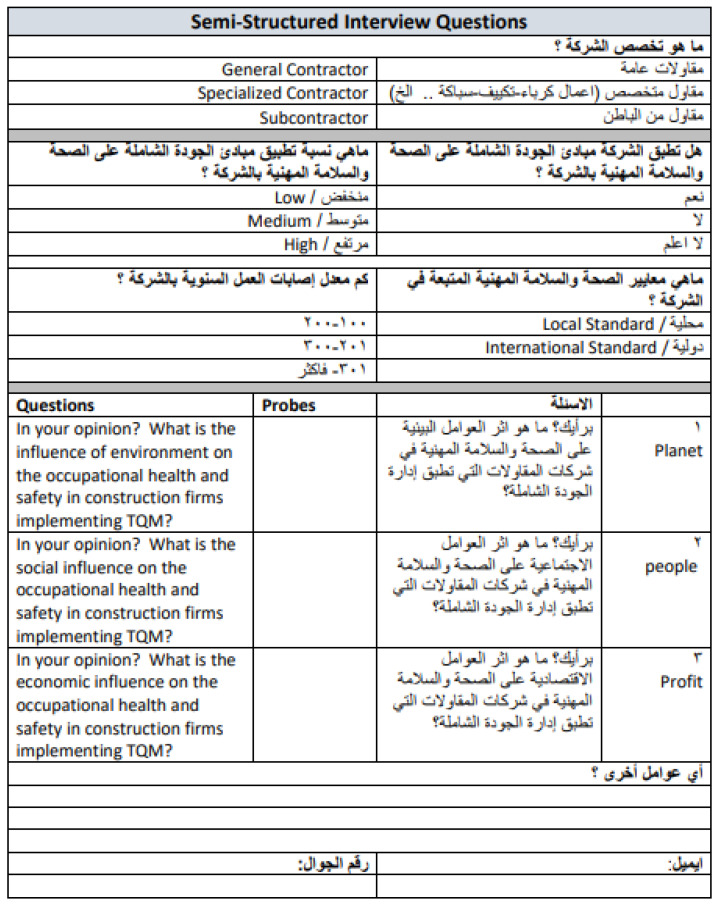
SSI questions including Arabic Language for non-English speaking participants, own study.

## Data Availability

Not applicable.
